# Supplementary figures and images for: First steps of bipedality in hominids: evidence from the atelid and proconsulid pelvis
Source: PeerJ. 2016 Jan 4;4:e1521. doi: 10.7717/peerj.1521 (PMC4715437; doi:10.7717/peerj.1521)

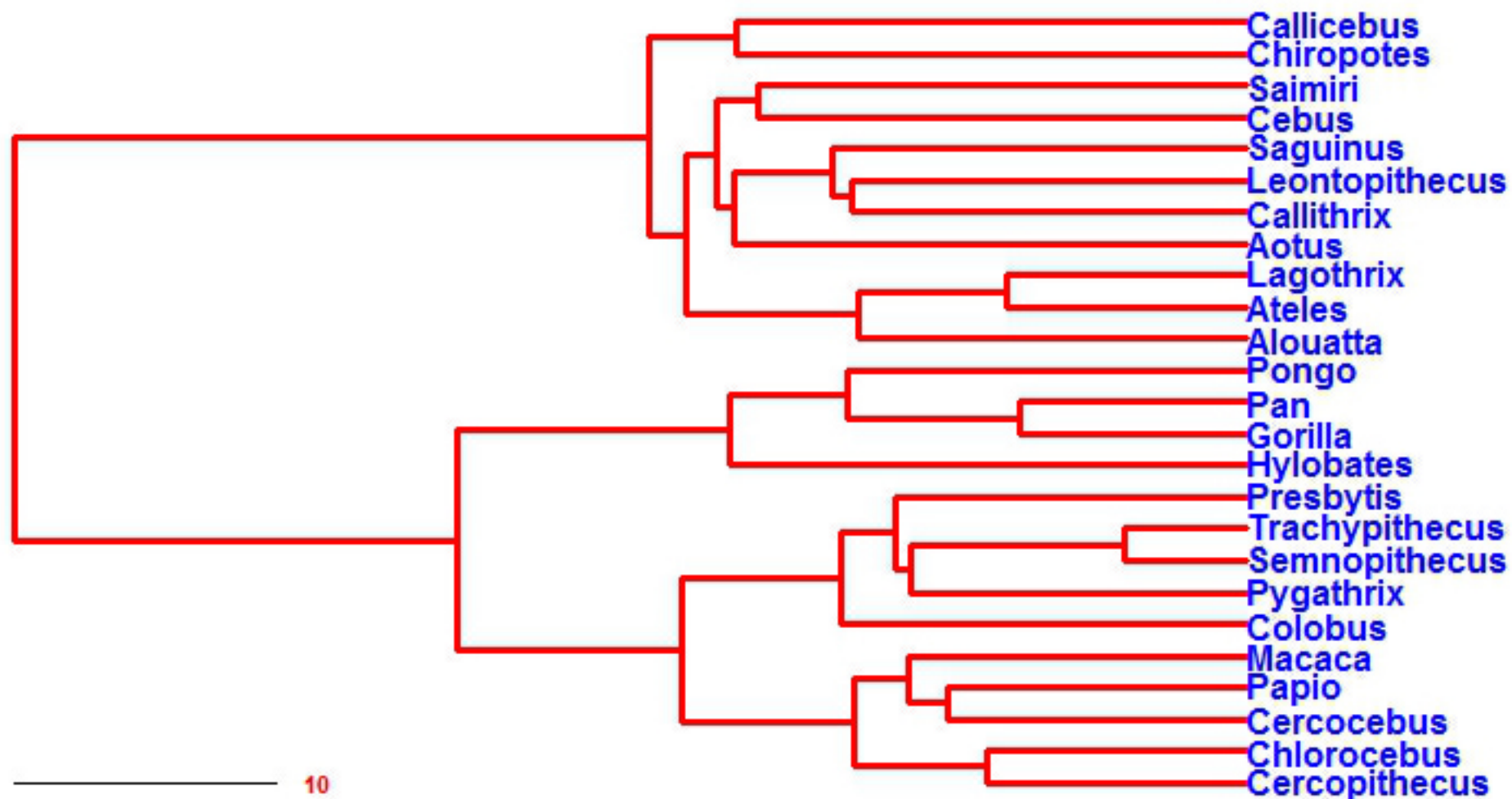

Supplement: Figure S1 — Generated from the 10KTrees project (Arnold, Matthews & Nunn, 2010). Scalebar = 10 millionyears. [file peerj-04-1521-s001.pdf]

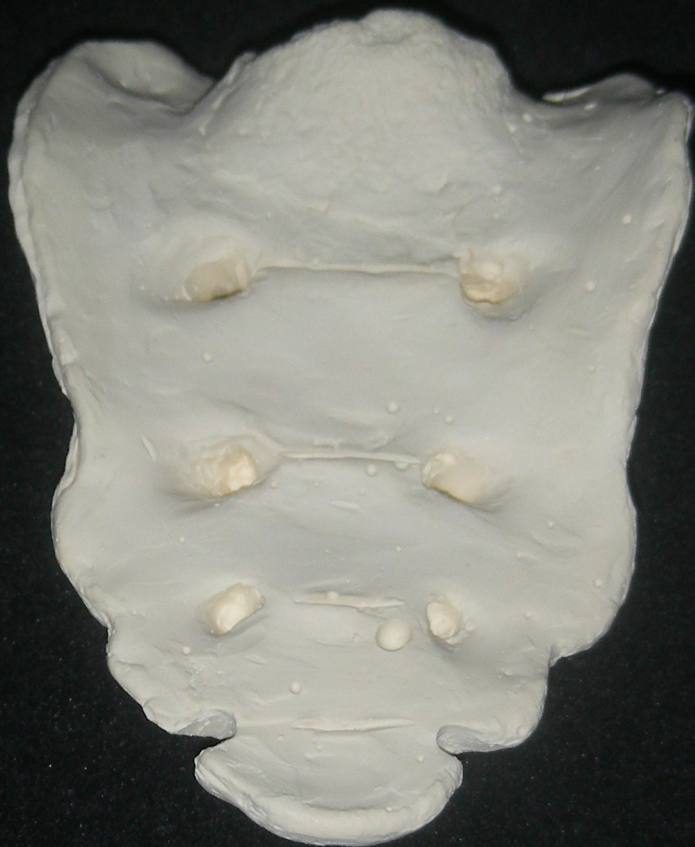

Supplement: Figure S2 — Part of the left ala from the original cast is obscured by the wax and clay to create a fit with innominate articular surface. [file peerj-04-1521-s002.pdf]

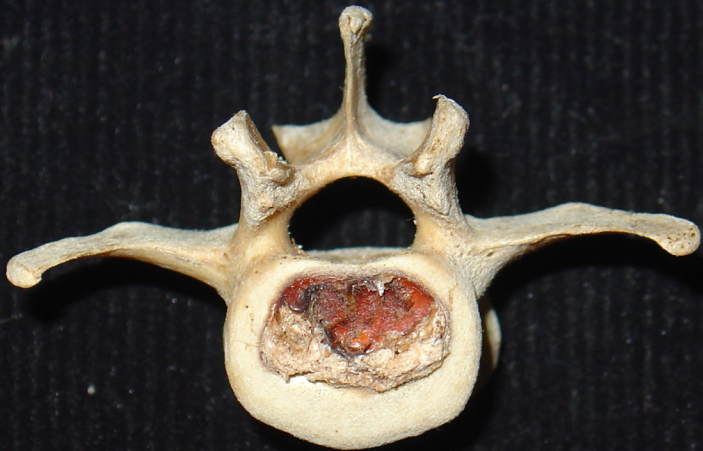

Supplement: Figure S3 — Note the position of the transverse processes at the base of the pedicle illustrating partial spinal invagination similar to other atelids (Figs.7). Specimen housed at the Museu Nacional de Rio de Janeiro (National Museum of Brazil). Photo credit: Sérgio L. Mendes. [file peerj-04-1521-s003.pdf]
