# Supplementary material for: First steps of bipedality in hominids: evidence from the atelid and proconsulid pelvis
Source: PeerJ. 2016 Jan 4;4:e1521. doi: 10.7717/peerj.1521 (PMC4715437; doi:10.7717/peerj.1521)
Supplement: Table S1 [file peerj-04-1521-s004.docx]

**Centrum Alar Iliac Iliac height**

**Species N width breadth height Acetabulum ratio**

*Alouatta guariba* 1 19.00 14.50 21.20 16.80 126.19

*Alouatta palliata* 8 17.93 (2.13) 10.76 (1.60) 20.84 (0.90) 16.76 (1.79) 125.21 (10.33)

*Alouatta caraya* 1 13.00 8.65 16.30 14.70 110.88

*Alouatta seniculus* 1 16.70 6.98 20.10 17.30 116.19

*Aloutta* sp. 1 22.80 11.20 19.90 17.20 115.70

*Ateles geoffroyi* 8 19.54 (0.85) 13.45 (2.12) 22.48 (2.36) 21.31 (1.89) 105.57 (7.60)

*Ateles* sp. 1 17.00 8.40 17.70 18.30 96.72

*Lagothrix lagotricha* 3 17.43 (0.71) 11.12 (2.24) 17.93 (1.70) 17.03 (0.80) 105.13 (5.53)

*Aotus vociferans* 1 9.60 6.55 7.10 6.90 102.90

*Callicebus* sp. 4 11.00 (1.09) 5.06 (0.81) 9.63 (1.80) 6.98 (1.50) 140.85 (30.99)

*Callithrix jacchus* 2 6.85 (0.92) 5.28 (0.67) 6.85 (0.78) 4.65 (0.35) 147.10 (5.54)

*Callithrix pygmaea* 2 4.65 (0.21) 4.98 (0.18) 6.10 (0.85) 3.35 (0.21) 183.26 (36.93)

*Cebus albifrons* 2 13.05 (1.06) 8.58 (1.24) 12.20 (0.00) 10.15 (0.64) 120.43 (7.55)

*Cebus capucinus* 4 14.55 (1.13) 11.33 (2.35) 24.48 (3.33) 11.60 (0.67) 210.38 (18.22)

*Cebus apella* 4 15.43 (1.94) 10.01 (2.02) 24.75 (2.77) 11.98 (1.33) 207.00 (12.54)

*Cebus* sp. 1 13.90 7.00 12.80 10.00 128.00

*Chiropotes santanas* 1 10.00 4.70 7.10 8.10 87.65

*Leontopithecus rosalia* 4 8.20 (0.61) 6.48 (0.83) 12.50 (2.11) 6.23 (0.78) 200.37 (16.54)

*Saguinus geoffroyi* 1 8.00 5.70 9.60 6.00 160.00

*Saguinus midas* 1 7.20 5.05 9.60 5.70 168.42

*Saguinus oedipus* 1 6.00 5.55 5.50 4.30 127.91

*Saimiri sciureus* 4 9.63 (0.95) 6.83 (0.71) 14.73 (0.33) 6.28 (0.29) 235.18 (15.32)

*Cercopithecus torquatus* 2 21.65 (1.63) 11.68 (1.17) 37.75 (3.61) 15.85 (0.92) 239.23 (36.63)

*Cercopithecus mona* 1 17.90 10.80 33.80 15.30 220.92

*Chlorocebus aethiops* 6 20.72 (2.09) 11.08 (1.04) 32.68 (7.05) 14.37 (2.35) 229.53 (40.74)

*Colobus guereza* 4 20.70 (4.02) 13.90 (2.28) 40.50 (4.83) 18.95 (3.44) 216.44 (25.66)

*Macaca fascicularis* 1 19.50 11.35 29.00 14.10 205.67

*Macaca mulatta* 1 19.80 7.70 25.60 14.00 182.86

*Macaca silenus* 1 21.50 13.05 36.70 18.60 197.31

*Papio hamadryas* 3 28.97 (0.51) 17.60 (0.54) 53.73 (5.67) 23.47 (1.63) 228.90 (17.62)

*Trachypithecus cristata* 1 18.40 8.90 31.40 14.60 215.07

*Presbytis rubicunda* 2 19.50 (0.99) 13.28 (0.32) 35.65 (7.99) 15.95 (1.63) 222.11 (27.45)

*Pygathrix* sp. 1 22.10 10.50 35.20 17.00 207.06

*Semnopithecus entellus* 1 25.50 15.35 38.10 18.30 208.20

*Theropithecus gelada* 2 28.65 (0.92) 15.88 (2.58) 48.15 (1.34) 21.35 (1.63) 226.42 (23.54)

*Trachypithecus pileatus* 1 20.60 12.00 36.30 17.30 209.83

*Homo sapiens* 20 48.85 (4.85) 32.69 (3.18) 40.66 (6.02) 52.06 (4.21) 78.43 (11.80)

*Pan troglodytes* 15 35.49 (3.51) 11.93 (3.20) 68.27 (7.16) 40.21 (2.96) 170.21 (17.15)

*Gorilla gorilla* 15 47.96 (6.15) 16.15 (3.53) 91.82 (16.60) 53.83 (6.67) 170.90 (24.79)

*Pongo pygmaeus* 7 34.17 (5.28) 12.38 (4.82) 61.83 (10.02) 37.40 (3.10) 165.38 (23.59)

*Hylobytes lar* 10 19.95 (1.25) 5.61 (1.02) 28.16 (3.46) 19.13 (2.37) 147.82 (13.68)

Fossil Specimens

KNM-MW 13142 (Ekembo reconst.) 30 17.7 – 18.7

A.L. 288-1 “Lucy” 34.4 28.5

KSD-VP-1/1 “Kadanuumuu” 43.8 33.5

Sts-14 27.0 24.5

BSN49/P27 36.0 24.5

Data will be provided upon request.
